# Differential immune cell densities in ductal carcinoma *In-Situ* and invasive breast cancer: Possible role of leukocytes in early stages of carcinogenesis

**DOI:** 10.12669/pjms.312.6481

**Published:** 2015

**Authors:** Bushra Sikandar, Muhammad Asif Qureshi, Talat Mirza, Saeed Khan, Lubna Avesi

**Affiliations:** 1 Dr. Bushra Sikandar, MBBS (Dow). M. Phil Student and Research Associate (Histopathology) Dow University of Health Sciences, Karachi – Pakistan; 2 Dr. Muhammad Asif Qureshi, MBBS, PhD Assistant Professor of Immunology, Department of Pathology, Dow University of Health Sciences, Karachi – Pakistan; 3 Dr. Talat Mirza, MBBS, M. Phil, PhD. Professor of Pathology, Dow University of Health Sciences, Karachi – Pakistan; 4 Dr. Saeed Khan, BSc, MSc, PhD, Post-Doc (USA). Assistant Professor of Pathology, Dow University of Health Sciences, Karachi – Pakistan; 5 Dr. Lunba Avesi, MBBS, FCPS. Assistant Professor of Pathology, Dow University of Health Sciences, Karachi – Pakistan

**Keywords:** Breast cancer, Immune cell densities, Immune infiltrates, Pre-neoplasia

## Abstract

**Objectives:**

To investigate immune cell densities in pre-neoplastic (DCIS), cancer (IDC) and control breast tissues.

**Methods:**

A total of four preneoplastic, 104 cancer and 104 control samples were analyzed. Morphological classification and prognostic scoring along with quantification of immune cells/mm^2^ was performed. Data were entered and analyzed using SPSS version 16. Correlation of immune cell densities with various tumour sub-types was investigated using paired t-test and ANOVA. A p-value of <0.05 was considered as significant.

**Results:**

Our data show increased infiltration of lymphocytes (mean lymphocytes = 287.6cells/mm^2^) as well as myelocytes (mean lymphocytes = 117.1cells/mm^2^) in pre-neoplastic tissues. This infiltration was significantly high compared to cancer (p-value<0.001) as well as control tissues (p-value <0.001). Moreover, we report increased infiltration of lymphocytes in cancer tissues compared to controls (p-value<0.001). There was no difference in lymphocyte densities within various tumour sub-types (all p-values >0.05).

**Conclusion:**

Leukocytes may play a role in early stages of breast carcinogenesis.

## INTRODUCTION

Breast Cancer ranks amongst the leading causes of death worldwide.^1^ According to the World Health Organization (WHO) it is estimated that mortality from breast cancer worldwide will continue to rise to 13.1 million in 2030.^2^ Breast cancer is emerging as the most prevalent malignancy in Asia including China, Japan, Taiwan, Singapore and India.^3^-^5^ In Pakistan breast cancer is the most common cancer in females with prevalence of 33.1% and age specific incidence rate of 51.7.^6^ Moreover, breast cancer has been reported to be amongst the leading causes of death in Pakistani females.^5^-^7^

Breast cancers can be classified using various parameters including histopathological sub-types, grade of the tumor, stage of the disease and receptor status.^8^,^9^ For prognostication of breast cancer, various parameters have been additionally used which include tumor size, tumor grade and lymph node status amongst others.^10^ The Nottingham prognostic index (NPI) is one of the standardized prognostic tool which is clinically useful, scientifically sound and widely reproducible.^11^,^12^

The NPI scoring system utilizes tumor size, histopathological grade and lymph node status and has been rated amongst the best prognostic tool available to date.^12^ However in the modern era of genomics and proteomics, the recent molecular profiling by microarray and immunohistochemistry detecting genetic signatures and receptor status are considered one of the utmost indicators towards targeted therapies.^13^ For example the 12^th^ St Gallen International Breast Conference guidelines classify breast cancer according to molecular intrinsic sub-typing. According to this classification system, breast cancers can be divided into Luminal A, Luminal B, Her-2 over expressing and triple negative (also called basal like) sub-types.^13^ Luminal A includes positive reactivity pattern of estrogen receptor (ER) and progesterone receptor (PgR), with low proliferative index (ki67), Luminal B includes ER and PgR positivity with high Ki67, Her-2 over expression showing strong positivity of Her-2 and absence of ER and PgR whereas the tripple negative sub-type (also known as Basal like) includes all cases which are neither ER, PgR positive nor expressing Her-2.^12^

Role of immune cells in breast carcinogenesis has been a matter of intense debate recently. It was previously believed that tumour infiltrating immune cells play a protective role in tumourigenesis. However, there is now increasing evidence to support the fact that the infiltrating immune cells play a role in carcinogenesis. Moreover, there is panoply of data to suggest strong links between chronic inflammation and carcinogenesis.^14^,^15^ This relationship is exemplified by chronic hepatitis B (HBV) infection and hepatocellular carcinoma, H pylori infection and gastric cancer and chemical irritants (for example smoke) with lung cancer.^16^ However, investigations into role of immune cells in breast carcinogenesis are scanty. Moreover, immune infiltrates have not been previously correlated with pre-neoplastic and neoplastic breast tissues.

In this study, we have quantified immune cell infiltration (based on microscopy performed on H & E stained tissues) in pre-neoplastic, neoplastic and control breast tissues. Moreover, immune cell densities have been correlated amongst pre-neoplastic, neoplastic and control breast tissues as well as within various tumour sub-types.

## METHODS

**Fig.1:**
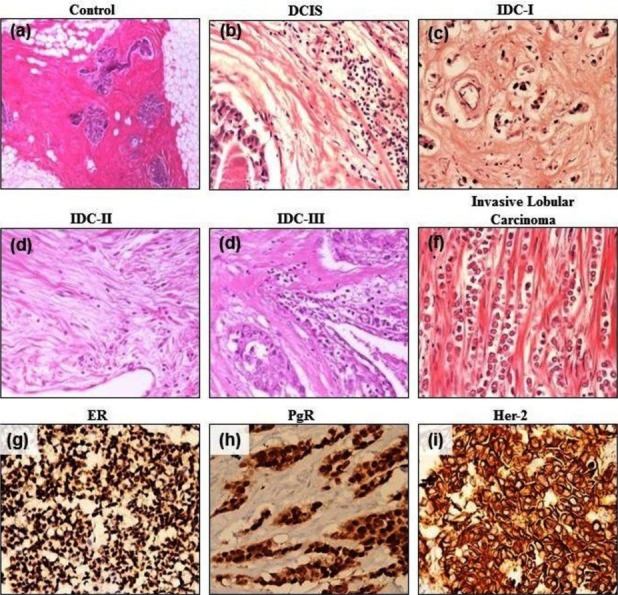
Haematoxylin and Eosin staining of Pre-neoplastic (DCIS), cancer (IDC) and control breast tissues. Images at ×400 magnification are shown. DCIS=ductal carcinoma in-situ; IDC=invasive ductal carcinoma; ER=estrogen receptor; PgR=progesterone receptor.

The study was conducted at the Dow University of Health Sciences after obtaining ethical approval from the Institutional Review Board, Ref # IRB-460/DUHS/14. A total of 04 pre-neoplastic (Ductal Carcinoma *In-Situ*=DCIS), 104 cancer mastectomies with lymph node dissection and 104 control tissues were analyzed in the study. Moreover, and in order to rule out any secondary impact on immune cell densities, samples with history of any inflammatory/infectious disorders were excluded from the study. In order to classify breast cancers using conventional and neoteric classification systems, all the breast cancer samples (n=104) were examined grossly to record their sizes and to dissect representative sections for staining. Lymph node involvement was also assessed using sections of lymph nodes for subsequent staining and microscopy. Moreover, all the pre-neoplastic (DCIS) and breast cancer samples were stained with Haematoxylin & Eosin for histological diagnosis and conventional grading (Fig.1 a-f). In order to classify breast cancer samples according to the molecular intrinsic sub-typing, antibodies against ER, PgR and Her-2 were used (Fig.1 g-i) and quantified using Allred scoring system.^16^ In order to score Her-2 staining, WHO guidelines were used.^16^ Moreover, in order to classify breast cancer tissues according to the NPI scoring system, morphology/grading, size of the tumour and lymph node status were determined.

In order to quantify immune cell densities (cells/mm^2^) in pre-neoplastic (DCIS), neoplastic (IDC) and control tissues, College of American Pathologists (CAP) guidelines were used.^16^ Immune cells were identified and grouped as either myelocyte cells or lymphoid cells based on morphology as observed via microscopy using H & E stained slides. Cell counting was performed at ×400 magnification at all times. Data were entered and analyzed using SPSS version 16.0.

## RESULTS

**Fig.2:**
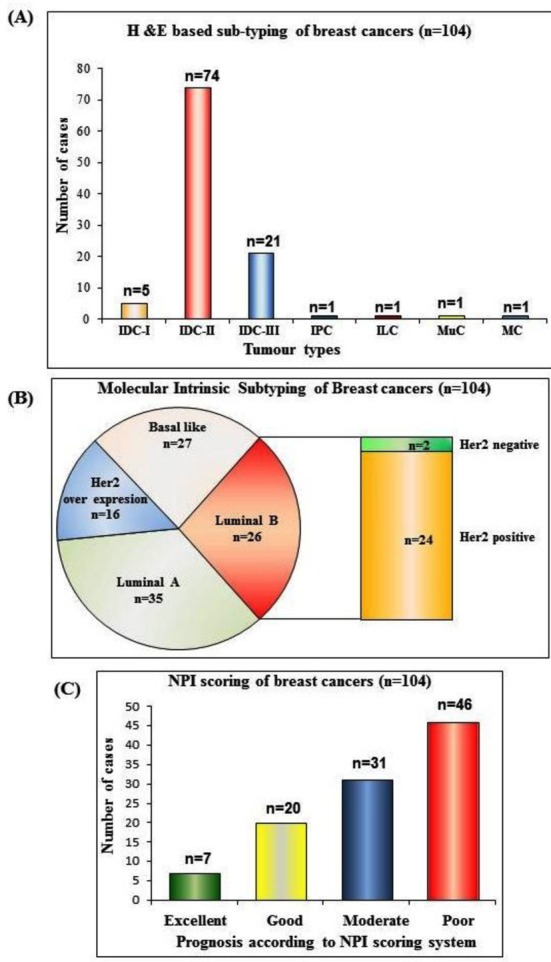
Classification of breast cancer using various classification systems. A= Classification of breast cancer according to conventional histopathological sub-typing, B= Classification of breast cancer according to molecular intrinsic sub-typing, C= Classification of breast cancer according to the NPI scoring system.

**Fig.3:**
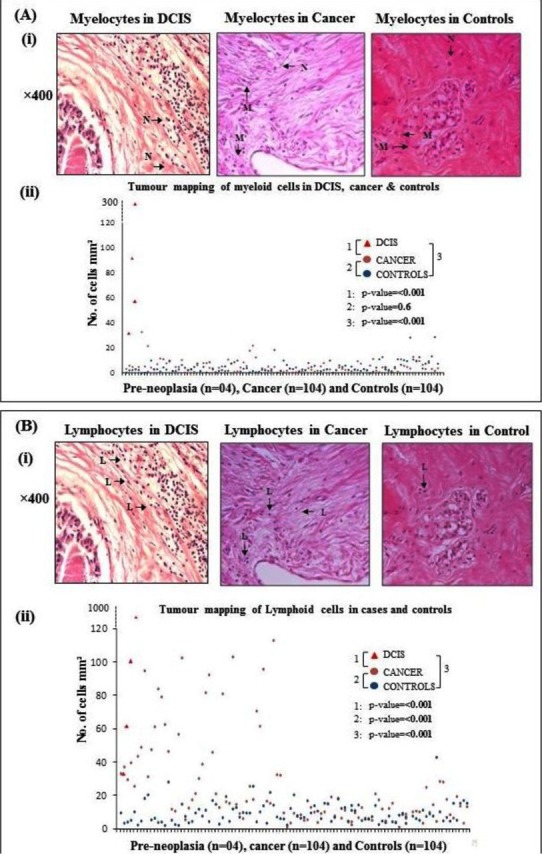
Immune cell densities in pre-neoplastic (DCIS), cancer (IDC) and control tissues. A= Tumour mapping of myelocytes in pre-neoplastic, cancer and control tissues, B= Tumour mapping of lymphocytes in pre-neoplastic, cancer and control tissues.

Frequencies of cancer samples according to various classification algorithms are demonstrated in Fig.2. Our investigations into quantification of immune cells demonstrate increased infiltration of both myeloid and lymphoid cells in pre-neoplastic (DCIS) tissues compared to control as well as cancer tissues (Table-I). Moreover, lymphocytes were the predominant immune cells in cancer (IDC) as well as control tissues (Table-I). However, there was significantly increased infiltration of lymphocytes in IDC tissues compared to controls (Fig.3), while there was no difference in myelocytes infiltration in IDC compared to control tissues. Taken together these data demonstrate increased infiltration of immune cells (both lymphocytes and myelocytes) in pre-neoplastic (DCIS) tissues compared to cancer (IDC) and control tissues. While in cancer tissues, there is increased infiltration of lymphocytes (and not myelocytes) compared to controls.

**Table-I:** Lymphocytic and Myelocytic infiltration in Pre-neoplastic (DCIS), cancer (IDC) and control tissues.

Immune cell densities within pre-neoplastic (DCIS), cancer (IDC) and control breast tissues						
	Controls		Pre-neoplasia (DCIS)		Cancer (IDC)	
	Mean cells/mm^2^	p-value	Mean cells/mm^2^	p-value	Mean cells/mm^2^	p-value
Lymphocytes	9.48	<0.001	286.7	0.48	25.35	<0.001
Myelocytes	5.04		117.1		5.35

Comparison of Lymphocytic and Myelocytic infiltration amongst pre-neoplastic (DCIS),cancer (IDC) and control breast tissues					
Tissue type	Lymphocytes (mean cells/mm^2^)	p-value	Tissue type	Myelocytes (mean cells/mm^2^)	p-value
IDC	25.35	<0.001	IDC	5.35	0.6
Controls	9.48		Controls	5.04	
DCIS	286.7	<0.001	DCIS	117.1	<0.001
IDC	25.35		IDC	5.35	
DCIS	286.7	<0.001	DCIS	117.1	<0.001
Controls	9.48		Controls	5.04	

In order to investigate correlation of immune cell densities in pre-neoplastic, invasive cancer tissue and various tumour sub-types, analyses of variance were undertaken. Our data demonstrates that immune cell infiltrates do not differ significantly amongst various tumour sub-types (Table-II). However, and interestingly, increased immune cell infiltration is observed in pre-neoplastic tissues (DCIS) compared to all tumour sub-types. Taken together, these data indicate increase immune cell infiltration in the early (pre-neoplastic) stages of carcinogenesis suggesting a role of these infiltrating cells in breast tumourigenesis at early stages.

**Table-II:** Correlation of immune cell infiltration amongst pre-neoplastic and various sub-types of breast cancer tissues.

Lymphocytes (cells/mm^2^)			Myelocytes (cells/mm^2^)		
Tumour types		p-value	Tumour types		p-value
Comparison of leukocytes in DCIS and invasive ductal carcinoma					
IDC-I	IDC-II	>0.99	IDC-I	IDC-II	0.2
	IDC-III	>0.99		IDC-III	0.67
	DCIS	<0.001		DCIS	<0.001
IDC-II	IDC-I	>0.99	IDC-II	IDC-I	0.2
	IDC-III	>0.99		IDC-III	>0.99
	DCIS	<0.001		DCIS	<0.001
IDC-III	IDC-I	>0.99	IDC-III	IDC-I	0.67
	IDC-II	>0.99		IDC-II	>0.99
	DCIS	<0.001		DCIS	<0.001

Comparison of leukocytes in DCIS and molecular intrinsic tumour sub-types					
Luminal A	Luminal B (Her2-)	>0.99	Luminal A	Luminal B (Her2-)	>0.99
	Luminal B (Her2+)	>0.99		Luminal B (Her2+)	>0.99
	Her2 ov.exp	>0.99		Her2 ov.exp	0.58
	Basal like	>0.99		Basal like	>0.99
	DCIS	<0.001		DCIS	<0.001
Luminal B (Her2-)	Luminal A	>0.99	Luminal B (Her2-)	Luminal A	>0.99
	Luminal B(Her2+)	>0.99		Luminal B(Her2+)	>0.99
	Her-2 ov.exp	>0.99		Her-2 ov.exp	>0.99
	Basal like	>0.99		Basal like	>0.99
	DCIS	0.003		DCIS	<0.001
Luminal B (Her2+)	Luminal A	>0.99	Luminal B (Her2+)	Luminal A	>0.99
	Luminal B (Her2-)	>0.99		Luminal B (Her2-)	>0.99
	Her2 ov. Exp.	0.53		Her2 ov. Exp.	>0.99
	Basal like	>0.99		Basal like	>0.99
	DCIS	<0.001		DCIS	<0.001
Her2 ov.exp.	Luminal A	>0.99	Her2 ov.exp.	Luminal A	0.58
	Luminal B (Her2-)	>0.99		Luminal B (Her2-)	>0.99
	Luminal B (Her2+)	0.53		Luminal B (Her2+)	>0.99
	Basal like	>0.99		Basal like	>0.99
	DCIS	<0.001		DCIS	<0.001
Basal like	Luminal A	>0.99	Basal like	Luminal A	>0.99
	Luminal B (Her2-)	>0.99		Luminal B (Her2-)	>0.99
	Luminal B (Her2+)	>0.99		Luminal B (Her2+)	>0.99
	Her2 ov. Exp.	>0.99		Her2 ov. Exp.	>0.99
	DCIS	<0.001		DCIS	<0.001

Comparision of leukocytes in DCIS and prognostic indices					
Excellent	Good	>0.99	Excellent	Good	>0.99
	Moderate	>0.99		Moderate	>0.99
	Poor	>0.99		Poor	>0.99
	DCIS	<0.001		DCIS	<0.001
Good	Excellent	>0.99	Good	Excellent	>0.99
	Moderate	>0.99		Moderate	>0.99
	Poor	>0.99		Poor	0.92
	DCIS	<0.001		DCIS	<0.001
Moderate	Excellent	>0.99	Moderate	Excellent	>0.99
	Good	>0.99		Good	>0.99
	Poor	>0.99		Poor	>0.99
	DCIS	<0.001		DCIS	<0.001
Poor	Excellent	>0.99	Poor	Excellent	>0.99
	Good	>0.99		Good	0.92
	Moderate	>0.99		Moderate	>0.99
	DCIS	<0.001		DCIS	<0.001

## DISCUSSION

Immune cell infiltration has been observed in a range of malignancies. However, exact mechanistic role (s) of these immune cells remains largely unclear. Investigations into the role leukocytes in various stages of tumourigenesis are scarce. In this study, we have investigated and quantified immune cells in pre-neoplastic (Ductal carcinoma *in-situ*=DCIS), neoplastic and control breast tissues. A noticeable edge of using pre-neoplastic samples is the fact that it allows us to investigate changes in immune cell densities in a sequential manner starting from pre-neopalstic stages through to neoplastic changes.

In this study, we show increased myelocyte and lymphocyte infiltration in the pre-neoplastic tissues compared to cancer and control tissues. In the invasive breast cancers, lymphocyte infiltration was significantly increased compared to control tissues. Moreover, lymphocyte infiltration when compared amongst different types of invasive cancers was not significantly different. Taken together, our data demonstrate that there is increased leukocyte infiltration in early stages (i.e. DCIS) of breast carcinogenesis. Once the carcinogenic events have commenced/settled (i.e. invasive cancers), a generalized increase in lymphocyte infiltration is observed that does not differ amongst various tumour sub-types.

In line with our findings, increased infiltration of lymphocytes in DCIS has been previously reported.^17^ Moreover, DCIS tumour microenvironment over-expresses various inflammatory mediators (probably released from infiltrating leukocytes), including interleukin signaling.^18^ Taken together, these findings suggest a role of leukocytes in early stages of breast carcinogenesis. In support of this notion, there is evidence that lymphocytes play a key role in creating a tumour promoting microenvironment.^14^ However, this need to be further investigated in breast carcinogenesis.

Another key finding in our study is heavy infiltration of lymphocytes (and not myelocytes) in invasive cancers compared to controls. It is possible that once the oncogenic processes (including genetic mutations, generation of inflammatory *milieu*) have commenced and settled; lymphocytes take over the charge and become predominant leukocytes in the breast cancer tissue. Moreover, lymphocyte densities do not differ amongst various breast tumour-subtypes. These findings are in line with the previously published reports showing increased infiltration of T-cells and B-cells in invasive breast carcinomas.^19^,^20^ However, what role these infiltrating lymphocytes are playing is under active investigation.

One could argue that the study has utilized only H & E staining to decipher immune cell densities. It is therefore important to mention here that identification of lymphocyte and myelocytes on H & E is a routine histological practice. However, H & E staining cannot differentiate between T and B lymphocytes (limitation of the present study). In order to delineate specific cell types, specific stains (such as CD-3 for T-cells and CD-19/CD-20 for B-cells) should be used.

## CONCLUSION

There is increased infiltration of both lymphocytes and myelocytes in pre-neoplastic breast tissues while invasive breast tissues are infiltrated with increase number of lymphocytes that do not vary within various breast sub-types.
